# Work factors facilitating working beyond state pension age: Prospective cohort study with register follow-up

**DOI:** 10.5271/sjweh.3904

**Published:** 2020-12-16

**Authors:** Lars L Andersen, Sannie V Thorsen, Mona Larsen, Emil Sundstrup, Cécile RL Boot, Reiner Rugulies

**Affiliations:** 1 National Research Centre for the Working Environment, Copenhagen, Denmark; 2 The Danish Center for Social Science Research, Copenhagen K, Denmark; 3 Department of Public and Occupational Health, VU University, Amsterdam UMC, Amsterdam, The Netherlands; 4 Department of Public Health, University of Copenhagen, Denmark; 5 Department of Psychology, University of Copenhagen, Denmark

**Keywords:** health, older worker, physical work demand, physical workload, psychosocial, retirement, statutory retirement age

## Abstract

**Objectives:**

The demographic changes in Europe underline the need for an extension of working lives. This study investigates the importance of physical work demands and psychosocial work factors for working beyond the state pension age (65 years).

**Methods:**

We combined data from three cohorts of the general working population in Denmark (DWECS 2005 and 2010, and DANES 2008), where actively employed workers aged 55–59 years replied to questionnaires about work environment and were followed until the age of 66 years in the Danish AMRun register of paid employment. Using logistic regression analyses, we calculated prevalence ratios (PR) and 95% confidence intervals (CI) for the association between physical and psychosocial work factors and working beyond state pension age, adjusted for age, sex, cohort, cohabiting, sector, income, vocational education, working hours, lifestyle, and previous sickness absence.

**Results:**

Of the 2884 workers aged 55–59 years, 1023 (35.5%) worked beyond the state pension age. Higher physical work demands was associated with a lower likelihood (PR 0.69, 95% CI 0.58–0.82) and a good psychosocial work environment was associated with higher likelihood (average of 7 items: PR 1.81, 95% CI 1.49–2.20) of working beyond state pension age. Stratified analyses did not change the overall pattern, ie, a good overall psychosocial work environment – as well as several specific psychosocial factors – increased the likelihood of working beyond state pension age, both for those with physically active and seated work.

**Conclusion:**

While high physical work demands was a barrier, a good psychosocial work environment seems to facilitate working beyond state pension age, also for those with physically active work.

In many Western societies, labor force participation rates have increased among individuals ≥60 years since the mid- or late-1990s (1). Increasing labor force participation rates are important in the light of the need in many countries of adapting to the increasing share of the population being 65 and older until the middle of this century. The main increase in older workers’ labor market participation has taken place before the state pension age, but working lives are also increasingly extended beyond this age (2). Extending work life beyond the state pension age can be a choice due to, eg, finding one’s job fulfilling, a necessity arising from lack of income as a retiree, or something in between (3, 4).

The state pension age is around 65 years in most EU countries (5). The propensity to work beyond this age differs to a large extent within the EU. The average employment rate in 2018 for the age group 65–74 years was 10.1%. However, this rate ranged from 2.7% in Luxembourg to 37.5% in Iceland (6). Working after the state pension age is less pronounced in Denmark (where the state pension age was 65 until the end of 2018) than in the other Scandinavian countries. Hence, the employment rate in 2018 for the 65–74-year-olds was 14.1% in Denmark compared to 16.8% in Sweden and 18.7% in Norway (7).

Working after the state pension age in Denmark is, presumably, to a lesser extent the result of necessity compared to many other countries, since the at-risk-of-poverty rate among individuals ≥65 years is fairly low in Denmark (8) – a result applying across gender and educational level due to fairly high replacement rates (ie, percentage of an individual’s annual employment income that is replaced by retirement income when they retire) for low income groups (9). In Denmark, only approximately 20% of working retirees – ie, those having paid work while also receiving pension – work in high-strain jobs. This is less than in many other European countries, eg, 30% in Sweden and between 40–50% in Estonia and Italy (4). Dingemans & Henkens (4) characterize high-strain jobs as unfavorable working conditions for older workers, namely full-time work, high physical and mental job demands, and low levels of job control. Their findings for working retirees suggest that participation in high-strain jobs is driven by necessity to a higher degree than participation in low-strain jobs, which is more likely to be a choice.

The present study considers the importance of good working conditions for working beyond the state retirement age when working is assumed to be mainly a choice. Therefore, the aim was first to investigate the role of physical and psychosocial work factors for working beyond the state pension age of 65 years. Further, distinguishing between two sub-groups, namely physically active jobs and seated jobs, the second aim was to explore whether the association between psychosocial work factors and working beyond the state retirement age differed by physical work characteristics. These analyses are critical for guiding employers in directing efforts towards retaining workers beyond the state pension age, in particular workers with physically active jobs, when work after this age is mainly a choice.

Most previous studies on working conditions and retirement have focused on retirement behavior in general or early retirement, while existing knowledge about working conditions that facilitate staying beyond the state pension age is more scarce and mainly based on cross-sectional or retrospective data (10). The results of these studies suggest that working conditions and psychological work environment are important determinants of working beyond the state pension age. Retrospective data (11) show that job control and work autonomy are associated with working after age 65 in Sweden, while having a physically or psychologically demanding job reduces the likelihood of working beyond this age. Similarly, using cross-sectional data from 16 European countries, the SHARE study reported that psychosocial working conditions were generally better among those working beyond retirement compared to previous conditions among those retired. In a review article distinguishing between work-related ‘facilitators’ and ‘barriers’ for a prolonged work life after pensionable age, working conditions tailored to individuals’ desire to contribute, flexible working hours, the possibility to upgrade existing and acquire new skills, and being offered financial gains are highlighted as facilitators, while barriers include stress, a lack of support, negative attitudes, physical and cognitive demands, and an overemphasis on lack of qualifications (12). Along the same line, a Finnish cohort study suggested that good mental health combined with the opportunity to control work time is a key factor in this respect (13). Finally, another Finnish cohort study showed that a higher likelihood of prolonging working life after state pension age among employees with higher occupational classes compared to lower occupational classes was explained by having physically light job, better work time control, and better self-rated work ability (14). Altogether, evidence from prospective studies are scarce.

The aim of this prospective cohort study with register follow-up was to investigate the role of physical and psychosocial work factors for working beyond the state pension age of 65 years. We hypothesized that physical work demands would act as a barrier and that positive psychosocial work factors would facilitate working beyond state pension age. As a secondary aim, we also explored whether the importance of psychosocial work factors were different for workers with physically active versus seated work.

This paper adds to the scare literature using prospective data to examine the role of physical and psychosocial work factors on working beyond the state pension age. The novelty of the paper is that separate but symmetric analyses are conducted on samples of older workers with physically active and seated work, respectively, providing for the first time, comparable estimates of the relative importance of a number of psychosocial work measures on working beyond the state pension age for these two groups of workers.

## Methods

### Study population

This study combines data of workers aged 55–59 years from three cohorts in Denmark (DWECS 2005 and 2010 and DANES 2008) (15–17), where actively employed workers replied to questionnaires about work environment and health. DWECS 2005 (N=19 855) and 2010 (N=31 210) were performed in the general working population and DANES 2008 (N=9913) in the general working population with an oversampling of those aged ≥50 years (N=4477, the sub-sample is called DANES 2008-senior). The response percentages of DWECS 2005 and 2010 and DANES 2008 were 63%, 53%, and 76%, respectively. Using the following five inclusion criteria, where each step adds to the previous criteria, the flow of participants was: (i) age 55–59 years and responding to the questionnaire (N=6079), (ii) actively employed at the time of the questionnaire response (N=5253), (iii) 66 years or older in 2018, i.e. we had access to AMRun register data (Danish: *Arbejdsmarkedsregnskab uden timenormering*) up until 2018 (N=3724), (iv) alive and not emigrated at the age of 66 years (N=3598), and (v) no missing covariates (N=2884). Thus, the final population for the present study consisted of 2884 workers aged 55–59 years at baseline.

### Predictors

Physical work demands was assessed with the single-item question *“How would you describe your physical activity at your main job?”* with four response categories (i) mostly sedentary work that does not require strenuous physical activity; (ii) mostly work while standing or walking but does not require strenuous physical activity; (iii) work while standing or walking with some lifting and carrying; and (iv) heavy or fast moving work that is physically strenuous (18).

For the psychosocial work factors, seven questions that were available in all three cohorts were included. All questions were originally developed to the Copenhagen Psychosocial Questionnaire (COPSOQ) (19, 20) and included (i) influence at work (*“Do you have a large degree of influence concerning your work?”*), (ii) workpace (*“Do you have to work very fast?”*), (iii) time to tasks (*“How often do you not have time to complete all your work tasks?”*), (iv) information about decisions (*“At your place at work, are you informed well in advance concerning for example important decisions, changes, or plans for the future?”*), (v) information to do well (*“Do you receive all the information you need in order to do your work well?”*), (vi) recognition from management (*“Is your work recognized and appreciated by the management?”*), and (vii) possibilities for development (*“Do you have the possibility of learning new things through your work?”*). The specific questions with response categories are provided in the supplementary material (www.sjweh.fi/show_abstract. php?abstract_id=3904), table S1.

### Normalization of predictor variables

For the predictor variables, physical work demands was linearly normalized on a scale of 0–1, ie, 0=seated work, 1/3=standing and walking at work, 2/3=lifting and carrying, and 1=heavy and fast. Response categories of the psychosocial variables were linearly normalized on a scale of 0–1, where 0 is worst and 1 is best.

### Outcome

The outcome variable was “working after state pension age” (yes/no), which was 65 years in Denmark until 2018. The AMRun register contains individual day-to-day information about labor market participation, unemployment, education, granted social benefits etc. of all citizens in Denmark (21). We defined working after state pension age as having any paid employment in the period from 65 years and 3 months to 66 years and 0 months. The reason for leaving out the first three months after turning 65 years is that there may be a short time lag from being eligible for state pension until actually leaving the labor market. With this definition, 35% of the participants in our study worked after the eligible state pension age.

### Control variables

The analyses were controlled for a number of factors that may influence the decision to work beyond state pension age; sex (man, woman), age at baseline (continuous variable 55–59 years), cohort (DWECS 2005, DWECS 2010, DANES 2008), cohabiting (married, cohabiting, single), sector (public sector, private sector), body mass index (BMI: underweight <18.5, normal weight 18.5–24.9, overweight 25–29.9 and obese ≥30 kg/m^2^), smoking status (never, former, current smoker), household disposable income (after tax, rent, and other fixed expenses) (0–15, 15–50, 50–85, 85–100 percentile; the 15th, 50th and 85th percentile are respectively 180 785, 263 031, and 367 067 Dkr, where 7.5 Dkr ~ 1 euro), weekly working hours (≤35, 35–40, >40 hours), vocational education [unskilled, skilled, and higher education (higher education includes short cycle higher educations, vocational bachelor educations, bachelor programs, masters programs, and PhD programs)], and long-term sickness absence within two years before baseline (yes, no). Information about sex, age, cohabitating, sector, family available income, vocational education, and previous long-term sickness absence were obtained at each respective baseline (2005, 2008 and 2010) based on registers from Statistic Denmark. Body weight and height (to subsequently calculate BMI), as well as smoking status and work hours were self-reported in the questionnaires.

### Statistics

Using log-binomial regression analysis (Proc Genmod, SAS version 9.4, SAS Institute, Cary NC, USA), we modelled the prevalence ratio (PR) for working after the state pension age as a function of each respective psychosocial work factor and physical work demands.

Estimating the PR from log-binomial regression – rather than the odds ratio from logistic regression – is recommended when the outcome is common (ie, >10%) (22). As most of the psychosocial factors were correlated with each other, we performed separate analysis for each factor to avoid multicollinearity. In model 1 (minimally adjusted), we adjusted for sex, baseline age, and cohort. In model 2 (fully adjusted), we adjusted for all the control variables mentioned before. As a next step, we analyzed in model 2 whether each of the psychosocial factors interacted with physical work demands, by including both the psychosocial factor and the physical activity at work factor in the same fully adjusted analysis, plus an interaction term between the particular psychosocial factor and the physical work demands (multiplicative interaction). For this purpose, the question about physical work demands was dichotomized, with ‘physically active work’ defined as the last three response categories (N=1595), and ‘seated work’ defined as the first response category (N=1248). We also performed an analysis with an overall measure of the psychosocial work environment, by first dichotomizing the 7 items to 0 or 1, then adding them together, and finally averaging by 7 (ie, normalized scale 0–1). Finally, we performed the analyses of each psychosocial factor as well as the overall measure of the psychosocial work environment stratified for physical work demands. In the fully adjusted model, we also tested for any interactions between the control variables ‘cohort’, ‘sex’ and ‘vocational education’ with each of the psychosocial work factors, the overall psychosocial work environment, and physical work demands. None of the interactions were statistically significant; hence we did not include the interaction terms in the final statistical models. Results are reported as PR and 95% CI. PR express the difference between 0 and 1 on the normalized scale.

## Results

Of the 2884 workers aged 55–59 years, 1023 (35.5%) worked beyond the state pension age of 65 years.

Table 1 shows the baseline characteristics of the 2884 workers. There were relative uniform distributions of men/women, DWECS/DANES cohort participants, normal weight/overweight participants, and public/private sector employees. Table 1 also illustrates baseline characteristics of those who retired at or before 65 years and those who continued to work beyond 65 years.

**Table 1:** Baseline characteristics of the workers aged 55–59 years of the study population.

Variable	All		Retired ≤65		Working >65	
		
	N	%	N	%	N	%
Sex						
Men	1330	46.1	711	38.2	619	60.5
Women	1554	53.9	1150	61.8	404	39.5
Cohort						
DWECS 2005	930	32.3	596	32.0	334	32.7
DWECS 2010	550	19.1	340	18.3	210	20.5
DANES 2008	1404	48.7	925	49.7	479	46.8
Cohabiting						
Married	2225	77.2	1445	77.7	780	76.3
Cohabiting	189	6.6	111	6.0	78	7.6
Single	470	16.3	305	16.4	165	16.1
Sector						
Public sector	1508	52.3	1018	54.7	490	47.9
Private sector	1376	47.7	843	45.3	533	52.1
Occupational education						
Unskilled	645	22.4	445	23.9	200	19.6
Skilled	1276	44.2	842	45.2	434	42.4
Higher education	963	33.4	574	30.8	389	38.0
Family income available (percentile)						
0–15	256	8.9	165	8.9	91	8.9
15–50	1028	35.6	700	37.6	328	32.1
50–85	1146	39.7	750	40.3	396	38.7
85–100	454	15.7	246	13.2	208	20.3
Working >65 years						
No	1861	64.5	1861	100	0	0
Yes	1023	35.5	0	0	1023	100
Weekly working hours						
≤35	809	28.1	612	32.9	197	19.3
35–40	1542	53.5	984	52.9	558	54.6
>40	533	18.5	265	14.2	268	26.2
Body mass index						
Underweight	33	1.1	23	1.2	10	1.0
Normal weight	1307	45.3	858	46.1	449	43.9
Overweight	1172	40.6	732	39.3	440	43.0
Obese	372	12.9	248	13.3	124	12.1
Smoking status						
Current smoker	1068	37.0	687	36.9	381	37.2
Ex-smoker	1059	36.7	658	35.4	401	39.2
Never	757	26.3	516	27.7	241	23.6
Long-term sickness absence the last 2 years before baseline						
No	2472	85.7	1555	83.6	917	89.6
Yes	412	14.3	306	16.4	106	10.4
Physical work demands						
Seated work	1248	43.9	725	39.5	523	51.9
Standing/walking	718	25.3	476	25.9	242	24.0
Lifting/carrying	750	26.4	549	29.9	201	19.9
Heavy/fast	127	4.5	85	4.6	42	4.2

Table 2 shows in the fully adjusted model that having higher physical work demands was associated with lower probability of working beyond retirement age (PR 0.69, 95% CI 0.58–0.82). In the fully adjusted model, all of the psychosocial work factors were associated with working beyond retirement age, with a PR range of 1.23–1.55. When combining the seven psychosocial factors (‘overall psychosocial work environment’), the PR was 1.81 (95% CI 1.49–2.20). Sex-stratified analyses showed largely similar results, although women tended to be more affected by high physical work demands than men (interaction: sex by physical work demands, P=0.07, sex-stratified PR in supplementary table S2).

**Table 2:** Association between physical and psychosocial work factors and working beyond state pension age. The prevalence ratios (PR) represent the highest value of each scale (reference: the lowest value), except for workpace which is reversed. [CI=confidence interval.]

Work factors	PR (95% CI)	

	Model 1^a^	Model 2 b
Physical		
Higher physical work demands	0.60 (0.51–0.70)	0.69 (0.58–0.82)
Psychosocial		
Influence at work	1.71 (1.42–2.06)	1.52 (1.25–1.84)
Workpace (low)	1.26 (1.04–1.53)	1.45 (1.19–1.77)
Time to tasks	1.07 (0.89–1.27)	1.23 (1.04–1.47)
Information about decisions	1.45 (1.20–1.76)	1.29 (1.07–1.57)
Information to do well	1.60 (1.28–2.00)	1.46 (1.17–1.84)
Recognition from management	1.57 (1.26–1.94)	1.48 (1.20–1.83)
Possibilities for development	1.83 (1.49–2.24)	1.55 (1.25–1.91)
Overall psychosocial work environment c	1.93 (1.59–2.35)	1.81 (1.49–2.20)

a Adjusted for age, sex, cohort.

b Adjusted for age, sex, cohort, cohabiting, sector, income, vocational education, working hours, lifestyle, and previous sickness absence.

c The ‘overall psychosocial work environment’ represents the average normalized 0–1 score of the other seven.

The interaction term between physical activity at work and psychosocial work environment was not statistically significant for any of the psychosocial variables. However, we still performed exploratory analyses to see if there were any marked numerical differences between the estimates. Table 3 shows the exploratory analysis with stratification for physical work demands. Four of the seven psychosocial work factors were significant for both for those with seated and physically active work. Thus, the stratified analyses did not change the overall picture.

**Table 3:** Association between psychosocial work factors and working beyond state pension age, stratified by physical activity at work. The prevalence ratios (PR) represent the highest value of each scale (reference: the lowest value), except for workpace which is reversed. [CI=confidence interval.]

Work factors	PR (95% CI)^a^		Interaction b (P-value)

	Seated work N=1248	Physically demanding work N=1595	
Influence at work	1.25 (0.95–1.66)	1.73 (1.31–2.29)	0.12
Workpace (low)	1.37 (1.04–1.80)	1.48 (1.10–1.98)	0.71
Time to tasks	1.21 (0.98–1.49)	1.46 (1.09–1.95)	0.35
Information about decisions	1.35 (1.04–1.75)	1.14 (0.85–1.53)	0.32
Information to do well	1.43 (1.06–1.95)	1.43 (1.01–2.02)	0.85
Recognition from management	1.50 (1.12–2.00)	1.40 (1.01–1.93)	0.59
Possibilities for development	1.35 (1.00–1.82)	1.56 (1.14–2.13)	0.69
Overall psychosocial work environment c	1.60 (1.25–2.05)	1.76 (1.31–2.36)	0.97

a Adjusted for age, sex, cohort, cohabiting, sector, income, vocational education, working hours, lifestyle, and previous sickness absence.

b The P-value of the interaction term between each psychosocial factor and physical activity at work is provided in the last column.

c The ‘overall psychosocial work environment’ represents the average normalized 0–1 score of the other seven.

## Discussion

In this prospective cohort study with register follow-up, we investigated the importance of physical work demands and psychosocial work factors for working beyond the state pension age. Higher physical work demands decreased the likelihood for continued work and can thus be considered a push factor. Higher influence, lower workpace, more time to complete tasks, more information about decisions, more information to do well, higher level of recognition from management and better possibilities for development increased the likelihood for working beyond retirement age. These psychosocial factors can thus be considered as stay factors. Subgroup analyses of those with seated and physically active work did not change the overall picture.

Previous studies have documented that high physical work demands act as a push factor (23–25). Thus, it was not surprising to find in the present study that those with a physically active work were only half as likely to work beyond the state pension age as those with seated work, even when adjusting for possible confounders. Poor health – eg, musculoskeletal pain – combined with high physical work demands likely plays an important role (26). Even at the same level of poor physical health, people with physically active work – eg, lifting, bending and twisting the back – are much more likely to be affected negatively in relation to being able to do their work (27). To break this barrier, workplaces may need to adapt the physical demands for those with physically active work who are still willing to work beyond the state pension age. The SeniorWorkingLife study showed that, among workers still active in the labor market, 25% of the men and 36% of the women with mainly physically active work would choose to stay longer in the labor market if the work was less physically strenuous (28).

As the most important finding, our results indicate that a good psychosocial work environment is associated with higher likelihood of work participation beyond the state pension age, even for those with physically active work. Previous studies on Dutch workers have shown mixed findings about the role of the psychosocial work environment; two studies on the same population of Dutch older workers showed significant associations between appreciation and interesting work and working beyond retirement (29, 30), although these associations were no longer present when health and work engagement were included in the analyses (29). A study on a prediction model for working beyond retirement showed that more procedural justice was associated with working beyond retirement, but again, the predictive power of health and sector of work was stronger in the final model (31).

### Strengths and limitations

A strength of the present study is its prospective design with reporting of the work environment several years before state pension age and follow-up in a high-quality Danish register until after the state pension age. By contrast, the majority of previous studies assessing important factors for working beyond retirement age has been cross-sectional questionnaire or qualitative interview studies. Another strength is that we controlled for a number of possible confounders that may influence the decision to work beyond the state pension age. Table 1 shows some clear differences at baseline between the groups, which highlights the need to control for possible confounders. However, there is a risk of over-adjustment when including income and vocational education in the analyses, as there may be some colinearity with the psychosocial factors and physical work demands. For this reason, we provided both a minimally and fully adjusted model, although the estimates of the fully adjusted model may be more conservative. A limitation is that we used single-item questions rather than full scales. However, these few and simple questions were associated with working beyond state pension age. The sample size is a limitation as it is difficult to detect possible statistical interactions with a relatively small sample of about 3000 participants. Thus, none of the interaction analyses were statistically significant, either because no relevant interaction exists or because the study was underpowered to detect such interactions. We provided exploratory stratified analyses of those with seated and physically active work, but these analyses should be interpreted with caution due to the sample size. While it would also be relevant to test associations for different levels of physical activity at work, the sample size in the present study is not large enough to allow for further stratification. Nevertheless, the overall picture is that the results hold true, also for those with physically active work. Another limitation is that we did not have information about the type of work that the participants did after the age of 65 years, ie, they may have changed to a less demanding job. A strength is that we controlled the analyses for previous sickness absence, as poor health is a strong predictor of early labor market exit. Nevertheless, we cannot exclude that the healthy worker effect influenced the findings. Furthermore, because the participants were of different ages, ie, 55–59 years, the follow-up time until the age of 66 years differed. However, controlling for age of the participants inherently accounts for this difference. Some of the control variables may be confounders whereas others may be effect modifiers. We tested for interactions of each of the psychosocial factors, the overall psychosocial work environment, and physical work demands with cohort, sex and vocational education. None of these showed significant interactions. To avoid the chance of mass significance, and because we did not have any specific hypotheses for doing so, we did not test for interactions with the remaining control variables.

### Concluding remarks

The present study investigated the importance of physical and psychosocial work factors for working beyond the state pension age. Higher physical work demands were a barrier for continued work and can thus be considered a push factor. By contrast, our result indicates that positive psychosocial work factors, such as high level of influence, recognition from the management, and possibilities for development can extend working life beyond state pension age, both among those with seated and physically active work. These can thus be considered stay factors.

## Supplementary material

Supplementary material
