# Supplementary material for: Work factors facilitating working beyond state pension age: Prospective cohort study with register follow-up
Source: Scand J Work Environ Health. 2020 Dec 16;47(1):15–21. doi: 10.5271/sjweh.3904 (PMC7801141; doi:10.5271/sjweh.3904)
Supplement: Supplementary material [file SJWEH-47-15-S001.pdf]

Work factors facilitating working beyond state pension age: Prospective cohort study with register follow-up [1]

by Lars L Andersen, PhD,[2] Sannie V Thorsen, PhD, Mona Larsen, PhD, Emil Sundstrup, PhD, Cécile RL Boot, PhD, Reiner Rugulies, PhD

1. *Supplementary material*
2. *Correspondence to: Lars L Andersen, National Research Centre for the Working Environment, Lersø Parkalle 105, DK-2100, Copenhagen, Denmark. [E-mail: lla@nfa.dk]*

Table S1. Specific questions and response categories

Predictor	Question	Response categories
Influence	Do you have a large degree of influence concerning your work?	Always; Often; Sometimes; Rarely; Never/Almost never
Workpace	Do you have to work very fast?	Always; Often; Sometimes; Rarely; Never/Almost never
Time to tasks	How often do you not have time to complete all your work tasks?	Always; Often; Sometimes; Rarely; Never/Almost never
Information about decisions	At your place at work, are you informed well in advance concerning for example important decisions, changes, or plans for the future	To a very high degree; To a high degree; To some degree; To a slight degree; To a very slight degree
information to do well	Do you receive all the information you need in order to do your work well?	To a very high degree; To a high degree; To some degree; To a slight degree; To a very slight degree
recognition from management	Is your work recognized and appreciated by the management?	To a very high degree; To a high degree; To some degree; To a slight degree; To a very slight degree
Possibilities for development	Do you have the possibility of learning new things through your work?	To a very high degree; To a high degree; To some degree; To a slight degree; To a very slight degree

Table S2. Sex-stratified analyses of Model 2 of Table 2. Association between physical and psychosocial work factors and working beyond state pension age for women and men, respectively. The prevalence ratios (PR) represent the highest value of each scale (reference: the lowest value), except for workpace which is reversed.

	**PR (95% CI)**	
	**Women**	**Men**
**Work factors**	**Model 2****	**Model 2****
Physical		
Higher physical work demands	0.52 (0.37 - 0.71)	0.78 (0.63 - 0.96)
Psychosocial		
Influence at work	1.41 (1.01 - 1.98)	1.53 (1.21 - 1.93)
Workpace (low)	1.67 (1.16 - 2.41)	1.41 (1.11 - 1.79)
Time to tasks	1.32 (0.96 - 1.83)	1.21 (0.99 - 1.48)

Information about decisions	1.60 (1.12 - 2.28)	1.24 (0.99 - 1.55)
Information to do well	1.81 (1.19 - 2.73)	1.35 (1.04 - 1.76)
Recognition from management	1.60 (1.10 - 2.33)	1.49 (1.15 - 1.93)
Possibilities for development	1.58 (1.07 - 2.33)	1.51 (1.17 - 1.95)
Overall psychosocial work environment[&]	1.76 (1.26 - 2.45)	1.82 (1.44 - 2.3)

**) Adjusted for age, sex, cohort, cohabiting, sector, income, vocational education, working hours, lifestyle, and previous sickness absence

&) The 'overall psychosocial work environment' represents the average normalized 0-1 score of the other seven.
